# Supplementary material for: Modulation of Canine Gut Microbiota by Prebiotic and Probiotic Supplements: A Long-Term In Vitro Study Using a Novel Colonic Fermentation Model
Source: Animals (Basel). 2024 Nov 20;14(22):3342. doi: 10.3390/ani14223342 (PMC11591024; doi:10.3390/ani14223342)
Supplement: Supplementary file 1 [file animals-14-03342-s001.zip › animals-3287654-Supplementary Figures.pdf]

Supplementary Figures

Figure S1: Absolute levels (log cells/mL) of different phyla in the luminal proximal colon (PC) at the end of the control (CTRL) and treatment (TR) period following treatment with the different test products (Microbiotal, M; probiotic, P; and their combination, M+P) (n=3). The intensity of the shading correlates with the absolute abundance, normalized for each of the different phyla (i.e. within each row).

Phylum	LUMEN					
	PC					
	MICROBIOTAL		PROBIOTIC		MICROBIOTAL + PROBIOTIC	
	CONTROL	TREATMENT	CONTROL	TREATMENT	CONTROL	TREATMENT
Actinobacteriota	7.94	8.26	7.99	7.76	8.05	8.31
Bacteroidota	7.50	7.98	7.23	7.56	7.64	7.78
Firmicutes	8.83	8.86	8.74	8.71	8.78	8.79
Proteobacteria	6.07	6.94	5.91	6.84	6.60	7.15

Figure S2: Absolute levels (log cells/mL) of different phyla in the luminal distal colon (DC) at the end of the control (CTRL) and treatment (TR) period following treatment with the different test products (Microbiotal, M; probiotic, P; and their combination, M+P) (n=3). The intensity of the shading correlates with the absolute abundance, normalized for each of the different phyla (i.e. within each row).

Phylum	LUMEN					
	DC					
	MICROBIOTAL		PROBIOTIC		MICROBIOTAL + PROBIOTIC	
	CONTROL	TREATMENT	CONTROL	TREATMENT	CONTROL	TREATMENT
Actinobacteriota	8.29	8.38	8.49	8.13	8.23	8.51
Bacteroidota	8.57	8.65	8.47	8.62	8.57	8.63
Firmicutes	9.12	9.12	9.07	9.08	9.05	9.10
Fusobacteriota	8.61	8.72	8.64	8.55	8.74	8.55
Proteobacteria	7.06	7.27	7.01	7.15	7.19	7.27

Figure S3: Proportional levels (%) of different phyla in the mucosal proximal colon (PC) at the end of the control (CTRL) and treatment (TR) period following treatment with the different test products (Microbiotal, M; probiotic, P; and their combination, M+P) (n=3). The intensity of the shading correlates with the absolute abundance, normalized for each of the different phyla (i.e. within each row).

Phylum	MUCUS					
	PC					
	MICROBIOTAL		PROBIOTIC		MICROBIOTAL + PROBIOTIC	
	CONTROL	TREATMENT	CONTROL	TREATMENT	CONTROL	TREATMENT
Actinobacteriota	16.4%	23.7%	19.6%	18.1%	11.6%	34.2%
Bacteroidota	18.6%	14.3%	10.9%	12.8%	15.2%	10.2%
Firmicutes	64.4%	61.6%	68.7%	68.1%	72.3%	55.3%
Proteobacteria	0.6%	0.3%	0.7%	0.9%	0.9%	0.3%

Figure S4: Proportional levels (%) of different phyla in the mucosal distal colon (DC) at the end of the control (CTRL) and treatment (TR) period following treatment with the different test products (Microbiotal, M; probiotic, P; and their combination, M+P) (n=3). The intensity of the shading correlates with the absolute abundance, normalized for each of the different phyla (i.e. within each row).

Phylum	MUCUS					
	DC					
	MICROBIOTAL		PROBIOTIC		MICROBIOTAL + PROBIOTIC	
	CONTROL	TREATMENT	CONTROL	TREATMENT	CONTROL	TREATMENT
Actinobacteriota	3.1%	2.6%	6.4%	1.3%	6.1%	6.7%
Bacteroidota	29.4%	30.1%	20.9%	23.0%	27.1%	23.9%
Firmicutes	25.4%	29.4%	28.9%	26.3%	39.2%	40.3%
Fusobacteriota	36.9%	33.0%	35.0%	36.6%	24.5%	26.7%
Proteobacteria	5.1%	4.9%	8.8%	12.9%	3.2%	2.4%

Figure S5: Absolute levels (log cells/mL) of different families in the luminal proximal colon (PC) at the end of the control (CTRL) and treatment (TR) period following treatment with the different test products (Microbiotal, M; probiotic, P; and their combination, M+P) (n=3). The intensity of the shading correlates with the absolute abundance, normalized for each of the different families (i.e. within each row).

Phylum	Family	LUMEN					
		PC					
		MICROBIOTAL		PROBIOTIC		MICROBIOTAL + PROBIOTIC	
		CONTROL	TREATMENT	CONTROL	TREATMENT	CONTROL	TREATMENT
Actinobacteriota	*Bifidobacteriaceae*	7.94	8.26	7.99	7.76	8.05	8.31
	Coriobacteriaceae	5.21	5.14	<LOQ	<LOQ	<LOQ	<LOQ
	Sanguibacteraceae	<LOQ	5.11	<LOQ	<LOQ	<LOQ	<LOQ
Bacteroidota	*Bacteroidaceae*	6.11	6.71	5.92	6.09	6.52	6.45
	Prevotellaceae	7.48	7.95	7.21	7.55	7.60	7.76
Firmicutes	*Acidaminococcaceae*	5.28	5.44	<LOQ	5.34	5.22	<LOQ
	Clostridiaceae	<LOQ	<LOQ	<LOQ	<LOQ	5.94	<LOQ
	Enterococcaceae	<LOQ	5.59	5.65	6.12	<LOQ	5.73
	Lachnospiraceae	6.39	6.39	6.26	6.25	6.56	6.45
	Lactobacillaceae	8.31	8.46	8.23	8.41	8.31	8.49
	Ruminococcaceae	6.43	6.27	6.24	6.57	6.60	5.56
	Selenomonadaceae	8.67	8.63	8.58	8.40	8.60	8.49
Proteobacteria	*Alcaligenaceae*	<LOQ	5.56	<LOQ	5.51	5.23	5.98
	Pseudomonadaceae	5.32	6.79	<LOQ	6.50	6.08	6.89
	Sutterellaceae	5.76	5.62	5.62	5.97	6.23	5.60
	Xanthomonadaceae	5.45	6.22	5.27	6.37	5.87	6.67

Figure S6: Absolute levels (log cells/mL) of different families in the luminal distal colon (DC) at the end of the control (CTRL) and treatment (TR) period following treatment with the different test products (Microbiotal, M; probiotic, P; and their combination, M+P) (n=3). The intensity of the shading correlates with the absolute abundance, normalized for each of the different families (i.e. within each row).

Phylum	Family	LUMEN					
		DC					
		MICROBIOTAL		PROBIOTIC		MICROBIOTAL + PROBIOTIC	
		CONTROL	TREATMENT	CONTROL	TREATMENT	CONTROL	TREATMENT
Actinobacteriota	*Bifidobacteriaceae*	8.29	8.38	8.49	8.13	8.23	8.51
	Coriobacteriaceae	6.30	6.21	6.00	5.98	5.71	5.95
	Eggerthellaceae	5.23	5.31	5.20	<LOQ	<LOQ	<LOQ
Bacteroidota	*Bacteroidaceae*	8.07	8.01	7.98	8.02	7.91	7.93
	Muribaculaceae	6.65	6.12	6.27	6.04	6.39	6.66
	Prevotellaceae	8.37	8.52	8.27	8.48	8.44	8.51
	Tannerellaceae	7.02	7.04	6.96	7.04	7.12	7.10
Firmicutes	*Acidaminococcaceae*	6.02	5.77	5.73	5.76	5.65	5.91
	Anaerovoracaceae	<LOQ	5.18	<LOQ	5.16	<LOQ	5.31
	Clostridiaceae	<LOQ	<LOQ	<LOQ	<LOQ	5.16	<LOQ
	Enterococcaceae	<LOQ	5.63	5.94	6.10	<LOQ	5.85
	Erysipelotrichaceae	<LOQ	5.49	<LOQ	5.89	5.56	<LOQ
	Lachnospiraceae	6.96	7.35	6.94	7.08	6.78	7.11
	Lactobacillaceae	8.25	8.17	8.36	8.50	8.28	8.41
	Lactobacillales_unclassified	<LOQ	<LOQ	5.27	5.15	<LOQ	<LOQ
	Oscillospiraceae	5.87	5.90	5.69	5.73	5.68	5.85
	Peptococcaceae	<LOQ	5.38	<LOQ	5.44	<LOQ	5.31
	Peptostreptococcaceae	6.26	6.87	6.36	6.96	6.29	6.53
	Ruminococcaceae	7.45	7.64	7.13	7.73	7.68	6.75
	Selenomonadaceae	8.90	8.77	8.87	8.78	8.78	8.88
	Streptococcaceae	8.48	8.71	8.21	8.29	8.44	8.36
Fusobacteriota	*Fusobacteriaceae*	8.61	8.72	8.64	8.55	8.74	8.55
Proteobacteria	*Alcaligenaceae*	<LOQ	5.42	<LOQ	5.12	5.32	5.57
	Erwiniaceae	5.30	<LOQ	<LOQ	<LOQ	<LOQ	<LOQ
	Pseudomonadaceae	5.64	6.66	5.48	5.90	5.75	6.48
	Sutterellaceae	7.02	7.06	6.98	7.09	7.14	7.08
	Xanthomonadaceae	5.55	6.27	5.26	5.67	5.79	6.48

Figure S7: Proportional levels (%) of different families in the mucosal proximal colon (PC) at the end of the control (CTRL) and treatment (TR) period following treatment with the different test products (Microbiotal, M; probiotic, P; and their combination, M+P) (n=3). The intensity of the s hading correlates with the absolute abundance, normalized for each of the different families (i.e. within each row).

Phylum	Family	MUCUS					
		PC					
		MICROBIOTAL		PROBIOTIC		MICROBIOTAL + PROBIOTIC	
		CONTROL	TREATMENT	CONTROL	TREATMENT	CONTROL	TREATMENT
Actinobacteriota	*Bifidobacteriaceae*	16.2%	23.6%	19.6%	18.1%	11.5%	34.1%
	Coriobacteriaceae	0.2%	0.1%	0.1%	0.1%	0.1%	0.1%
Bacteroidota	*Bacteroidaceae*	3.4%	2.1%	4.6%	4.1%	6.0%	2.7%
	Prevotellaceae	15.2%	12.2%	6.3%	8.8%	9.2%	7.5%
Firmicutes	*Acidaminococcaceae*	0.0%	0.1%	0.1%	0.1%	0.1%	0.0%
	Clostridiaceae	0.1%	0.0%	0.0%	0.0%	0.1%	0.0%
	Enterococcaceae	0.0%	0.0%	0.1%	0.0%	0.0%	0.0%
	Erysipelotrichaceae	0.0%	0.3%	0.1%	0.4%	0.4%	0.1%
	Lachnospiraceae	1.6%	1.4%	1.3%	1.4%	2.3%	0.5%
	Lactobacillaceae	15.3%	27.4%	13.4%	23.8%	12.3%	20.4%
	Oscillospiraceae	0.0%	0.0%	0.0%	0.0%	0.1%	0.0%
	Ruminococcaceae	3.1%	2.3%	4.6%	6.8%	3.7%	3.0%
	Selenomonadaceae	44.2%	30.0%	49.1%	35.5%	53.4%	31.2%
Proteobacteria	*Sutterellaceae*	0.6%	0.3%	0.7%	0.8%	0.9%	0.2%
	Xanthomonadaceae	0.0%	0.0%	0.0%	0.1%	0.0%	0.0%

Figure S8: Proportional levels (%) of different families in the mucosal distal colon (DC) at the end of the control (CTRL) and treatment (TR) period following treatment with the different test products (Microbiotal, M; probiotic, P; and their combination, M+P) (n=3). The intensity of the shading correlates with the absolute abundance, normalized for each of the different families (i.e. within each row).

Phylum	Family	MUCUS					
		DC					
		MICROBIOTAL		PROBIOTIC		MICROBIOTAL + PROBIOTIC	
		CONTROL	TREATMENT	CONTROL	TREATMENT	CONTROL	TREATMENT
Actinobacteriota	*Bifidobacteriaceae*	2.4%	2.1%	5.8%	0.8%	5.1%	6.1%
	Coriobacteriaceae	0.7%	0.5%	0.6%	0.4%	1.0%	0.7%
Bacteroidota	*Bacteroidaceae*	13.5%	15.7%	10.5%	10.2%	12.4%	10.3%
	Muribaculaceae	0.5%	0.4%	0.5%	0.6%	0.8%	1.1%
	Prevotellaceae	1.5%	1.3%	1.6%	1.5%	1.6%	2.3%
	Tannerellaceae	14.0%	12.6%	8.3%	10.6%	12.4%	10.2%
Firmicutes	*Acidaminococcaceae*	0.1%	0.0%	0.1%	0.0%	0.0%	0.0%
	Anaerovoracaceae	0.2%	0.1%	0.1%	0.1%	0.2%	0.1%
	Clostridiaceae	0.1%	0.0%	0.0%	0.0%	0.1%	0.0%
	Erysipelotrichaceae	0.1%	0.3%	0.4%	0.7%	0.3%	0.4%
	Family_XI	0.0%	1.0%	0.0%	0.0%	0.0%	0.0%
	Lachnospiraceae	7.7%	9.7%	5.4%	6.8%	7.6%	9.6%
	Lactobacillaceae	1.2%	1.3%	1.6%	1.1%	2.5%	2.1%
	Oscillospiraceae	0.8%	1.2%	0.6%	0.8%	0.7%	1.5%
	Peptostreptococcaceae	0.6%	0.5%	0.6%	0.8%	1.1%	1.2%
	Ruminococcaceae	0.2%	0.2%	0.1%	0.3%	0.5%	0.2%
	Selenomonadaceae	10.5%	5.7%	16.4%	10.9%	19.2%	9.2%
	Streptococcaceae	3.9%	9.2%	3.5%	4.7%	6.8%	15.8%
Fusobacteriota	*Fusobacteriaceae*	36.9%	33.0%	35.0%	36.6%	24.5%	26.7%
Proteobacteria	*Enterobacteriaceae*	0.0%	0.0%	0.0%	0.1%	0.0%	0.0%
	Succinivibrionaceae	2.9%	3.5%	6.7%	10.5%	0.4%	0.3%
	Sutterellaceae	2.1%	1.4%	2.1%	2.2%	2.7%	2.1%

Figure S9: Absolute levels (log cells/mL) of the 25 most abundant genera in the luminal proximal colon (PC) at the end of the control (CTRL) and treatment (TR) period following treatment with the different test products (Microbiotal, M; probiotic, P; and their combination, M+P) (n=3). The intensity of the shading correlates with the absolute abundance, normalized for each of the different genera (i.e. within each row).

Phylum	Family	Genus	LUMEN					
			PC					
			MICROBIOTAL		PROBIOTIC		MICROBIOTAL + PROBIOTIC	
			CONTROL	TREATMENT	CONTROL	TREATMENT	CONTROL	TREATMENT
Actinobacteriota	*Bifidobacteriaceae*	*Bifidobacterium*	7.94	8.26	7.99	7.76	8.05	8.31
	Coriobacteriaceae	*Collinsella*	5.21	5.14	<LOQ	<LOQ	<LOQ	<LOQ
Bacteroidota	*Bacteroidaceae*	*Bacteroides*	6.11	6.71	5.92	6.09	6.52	6.45
	Muribaculaceae	*Muribaculaceae_ge*	<LOQ	<LOQ	<LOQ	<LOQ	<LOQ	<LOQ
	Prevotellaceae	*Alloprevotella*	6.55	7.25	5.60	5.94	6.83	5.89
		Prevotella_9	7.43	7.86	7.20	7.54	7.52	7.76
	Tannerellaceae	*Parabacteroides*	<LOQ	<LOQ	<LOQ	<LOQ	<LOQ	<LOQ
Firmicutes	*Erysipelotrichaceae*	*Holdemanella*	<LOQ	<LOQ	<LOQ	<LOQ	<LOQ	<LOQ
	Lachnospiraceae	*Blautia*	<LOQ	<LOQ	<LOQ	<LOQ	<LOQ	<LOQ
		Hungatella	<LOQ	<LOQ	<LOQ	<LOQ	<LOQ	<LOQ
		Lachnoclostridium	6.28	6.31	6.21	6.21	6.51	6.37
		Lachnospiraceae_ge	<LOQ	<LOQ	<LOQ	<LOQ	<LOQ	<LOQ
		Lachnospiraceae_unclassified	5.72	5.63	5.27	5.22	5.59	5.57
	Lactobacillaceae	*Lactobacillus*	8.29	8.38	8.21	8.32	8.26	8.40
		Limosilactobacillus	6.98	7.71	6.91	7.69	7.30	7.73
	Oscillospiraceae	*Intestinimonas*	<LOQ	<LOQ	<LOQ	<LOQ	<LOQ	<LOQ
	Peptostreptococcaceae	*Peptoclostridium*	<LOQ	<LOQ	<LOQ	<LOQ	<LOQ	<LOQ
	Ruminococcaceae	*Faecalibacterium*	6.43	6.27	6.24	6.57	6.60	5.56
	Selenomonadaceae	*Megamonas*	8.67	8.63	8.58	8.40	8.60	8.49
	Streptococcaceae	*Streptococcus*	<LOQ	<LOQ	<LOQ	<LOQ	<LOQ	<LOQ
Fusobacteriota	*Fusobacteriaceae*	*Fusobacterium*	<LOQ	<LOQ	<LOQ	<LOQ	<LOQ	<LOQ
Proteobacteria	*Pseudomonadaceae*	*Pseudomonas*	5.32	6.79	<LOQ	6.50	6.08	6.89
	Succinivibrionaceae	*Anaerobiospirillum*	<LOQ	<LOQ	<LOQ	<LOQ	<LOQ	<LOQ
	Sutterellaceae	*Sutterella*	5.76	5.62	5.62	5.97	6.23	5.60
	Xanthomonadaceae	*Stenotrophomonas*	5.45	6.22	5.27	6.37	5.87	6.67

Figure S10: Absolute levels (log cells/mL) of the 25 most abundant genera in the luminal distal colon (DC) at the end of the control (CTRL) and treatment (TR) period following treatment with the different test products (Microbiotal, M; probiotic, P; and their combination, M+P) (n=3). The intensity of the shading correlates with the absolute abundance, normalized for each of the different genera (i.e. within each row).

Phylum	Family	Genus	LUMEN					
			DC					
			MICROBIOTAL		PROBIOTIC		MICROBIOTAL + PROBIOTIC	
			CONTROL	TREATMENT	CONTROL	TREATMENT	CONTROL	TREATMENT
Actinobacteriota	*Bifidobacteriaceae*	*Bifidobacterium*	8.29	8.38	8.49	8.13	8.23	8.51
	Coriobacteriaceae	*Collinsella*	6.30	6.21	6.00	5.98	5.71	5.95
Bacteroidota	*Bacteroidaceae*	*Bacteroides*	8.07	8.01	7.98	8.02	7.91	7.93
	Muribaculaceae	*Muribaculaceae_ge*	6.65	6.12	6.27	6.04	6.39	6.66
	Prevotellaceae	*Alloprevotella*	8.30	8.36	8.18	8.41	8.35	8.33
		Prevotella_9	7.53	8.00	7.53	7.64	7.68	8.05
	Tannerellaceae	*Parabacteroides*	7.02	7.04	6.96	7.04	7.12	7.10
Firmicutes	*Erysipelotrichaceae*	*Holdemanella*	<LOQ	<LOQ	<LOQ	5.38	5.30	<LOQ
	Lachnospiraceae	*Blautia*	6.07	6.83	6.21	6.47	5.97	6.56
		Hungatella	5.49	5.64	<LOQ	<LOQ	<LOQ	<LOQ
		Lachnoclostridium	6.57	6.77	6.61	6.65	6.43	6.67
		Lachnospiraceae_ge	5.19	5.35	<LOQ	5.53	5.17	<LOQ
		Lachnospiraceae_unclassified	6.53	6.92	6.44	6.60	6.31	6.63
	Lactobacillaceae	*Lactobacillus*	8.24	8.13	8.35	8.47	8.27	8.38
		Limosilactobacillus	6.48	7.04	6.51	7.29	6.73	7.24
	Oscillospiraceae	*Intestinimonas*	5.71	5.74	5.60	5.48	5.52	5.71
	Peptostreptococcaceae	*Peptoclostridium*	6.24	6.87	6.35	6.95	6.27	6.52
	Ruminococcaceae	*Faecalibacterium*	7.43	7.63	7.11	7.73	7.68	6.72
	Selenomonadaceae	*Megamonas*	8.90	8.77	8.87	8.78	8.78	8.88
	Streptococcaceae	*Streptococcus*	8.48	8.71	8.21	8.29	8.44	8.36
Fusobacteriota	*Fusobacteriaceae*	*Fusobacterium*	8.61	8.72	8.64	8.55	8.74	8.55
Proteobacteria	*Pseudomonadaceae*	*Pseudomonas*	5.64	6.66	5.48	5.90	5.75	6.48
	Succinivibrionaceae	*Anaerobiospirillum*	<LOQ	<LOQ	<LOQ	<LOQ	<LOQ	<LOQ
	Sutterellaceae	*Sutterella*	7.01	7.06	6.97	7.09	7.14	7.08
	Xanthomonadaceae	*Stenotrophomonas*	5.55	6.27	5.26	5.67	5.79	6.48

Figure S11: Proportional levels (%) of the 25 most abundant genera in the mucosal proximal colon (PC) at the end of the control (CTRL) and treatment (TR) period following treatment with the different test products (Microbiotal, M; probiotic, P; and their combination, M+P) (n=3). The intensity of the shading correlates with the absolute abundance, normalized for each of the different genera (i.e. within each row).

Phylum	Family	Genus	MUCUS					
			PC					
			MICROBIOTAL		PROBIOTIC		MICROBIOTAL + PROBIOTIC	
			CONTROL	TREATMENT	CONTROL	TREATMENT	CONTROL	TREATMENT
Actinobacteriota	*Bifidobacteriaceae*	*Bifidobacterium*	16.2%	23.6%	19.6%	18.1%	11.5%	34.1%
	Coriobacteriaceae	*Collinsella*	0.2%	0.1%	0.1%	0.1%	0.1%	0.1%
Bacteroidota	*Bacteroidaceae*	*Bacteroides*	3.4%	2.1%	4.6%	4.1%	6.0%	2.7%
	Muribaculaceae	*Muribaculaceae_ge*	0.0%	0.0%	0.0%	0.0%	0.0%	0.0%
	Prevotellaceae	*Alloprevotella*	14.2%	10.2%	5.5%	7.3%	8.4%	4.3%
		Prevotella_9	1.0%	2.1%	0.8%	1.5%	0.7%	3.2%
	Tannerellaceae	*Parabacteroides*	0.0%	0.0%	0.0%	0.0%	0.0%	0.0%
Firmicutes	*Erysipelotrichaceae*	*Holdemanella*	0.0%	0.3%	0.1%	0.4%	0.3%	0.1%
	Lachnospiraceae	*Blautia*	0.0%	0.0%	0.0%	0.0%	0.0%	0.0%
		Hungatella	0.0%	0.0%	0.0%	0.0%	0.0%	0.0%
		Lachnoclostridium	0.7%	0.4%	0.7%	0.4%	1.2%	0.2%
		Lachnospiraceae_ge	0.0%	0.0%	0.0%	0.0%	0.0%	0.0%
		Lachnospiraceae_unclassified	0.9%	1.0%	0.6%	0.9%	1.1%	0.3%
	Lactobacillaceae	*Lactobacillus*	14.1%	24.9%	12.0%	20.1%	11.1%	16.7%
		Limosilactobacillus	1.2%	2.5%	1.4%	3.8%	1.2%	3.7%
	Oscillospiraceae	*Intestinimonas*	0.0%	0.0%	0.0%	0.0%	0.0%	0.0%
	Peptostreptococcaceae	*Peptoclostridium*	0.0%	0.0%	0.0%	0.0%	0.0%	0.0%
	Ruminococcaceae	*Faecalibacterium*	3.1%	2.3%	4.6%	6.8%	3.7%	3.0%
	Selenomonadaceae	*Megamonas*	44.2%	30.0%	49.1%	35.5%	53.4%	31.2%
	Streptococcaceae	*Streptococcus*	0.0%	0.0%	0.0%	0.0%	0.0%	0.0%
Fusobacteriota	*Fusobacteriaceae*	*Fusobacterium*	0.0%	0.0%	0.0%	0.0%	0.0%	0.0%
Proteobacteria	*Pseudomonadaceae*	*Pseudomonas*	0.0%	0.0%	0.0%	0.0%	0.0%	0.0%
	Succinivibrionaceae	*Anaerobiospirillum*	0.0%	0.0%	0.0%	0.0%	0.0%	0.0%
	Sutterellaceae	*Sutterella*	0.6%	0.3%	0.7%	0.8%	0.9%	0.2%
	Xanthomonadaceae	*Stenotrophomonas*	0.0%	0.0%	0.0%	0.1%	0.0%	0.0%

Figure S12: Proportional levels (%) of the 25 most abundant genera in the mucosal distal colon (DC) at the end of the control (CTRL) and treatment (TR) period following treatment with the different test products (Microbiotal, M; probiotic, P; and their combination, M+P) (n=3). The intensity of the shading correlates with the absolute abundance, normalized for each of the different genera (i.e. within each row).

Phylum	Family	Genus	MUCUS					
			DC					
			MICROBIOTAL		PROBIOTIC		MICROBIOTAL + PROBIOTIC	
			CONTROL	TREATMENT	CONTROL	TREATMENT	CONTROL	TREATMENT
Actinobacteriota	*Bifidobacteriaceae*	*Bifidobacterium*	2.4%	2.1%	5.8%	0.8%	5.1%	6.1%
	Coriobacteriaceae	*Collinsella*	0.7%	0.5%	0.6%	0.4%	1.0%	0.7%
Bacteroidota	*Bacteroidaceae*	*Bacteroides*	13.5%	15.7%	10.5%	10.2%	12.4%	10.3%
	Muribaculaceae	*Muribaculaceae_ge*	0.5%	0.4%	0.5%	0.6%	0.8%	1.1%
	Prevotellaceae	*Alloprevotella*	1.4%	1.3%	1.6%	1.4%	1.5%	2.1%
		Prevotella_9	0.0%	0.1%	0.0%	0.1%	0.0%	0.2%
	Tannerellaceae	*Parabacteroides*	14.0%	12.6%	8.3%	10.6%	12.4%	10.2%
Firmicutes	*Erysipelotrichaceae*	*Holdemanella*	0.1%	0.1%	0.3%	0.6%	0.3%	0.4%
	Lachnospiraceae	*Blautia*	0.6%	1.8%	0.6%	1.9%	1.1%	2.4%
		Hungatella	1.1%	1.5%	0.3%	0.4%	0.3%	0.6%
		Lachnoclostridium	2.4%	2.1%	2.4%	1.9%	2.4%	2.6%
		Lachnospiraceae_ge	0.3%	0.1%	0.2%	0.2%	0.9%	1.0%
		Lachnospiraceae_unclassified	3.2%	4.0%	1.9%	2.3%	2.8%	2.8%
	Lactobacillaceae	*Lactobacillus*	1.1%	1.3%	1.5%	1.0%	2.4%	2.0%
		Limosilactobacillus	0.1%	0.0%	0.1%	0.1%	0.1%	0.2%
	Oscillospiraceae	*Intestinimonas*	0.5%	0.9%	0.4%	0.6%	0.6%	1.3%
	Peptostreptococcaceae	*Peptoclostridium*	0.5%	0.5%	0.5%	0.8%	1.0%	1.1%
	Ruminococcaceae	*Faecalibacterium*	0.1%	0.1%	0.1%	0.2%	0.2%	0.1%
	Selenomonadaceae	*Megamonas*	10.5%	5.7%	16.4%	10.9%	19.2%	9.2%
	Streptococcaceae	*Streptococcus*	3.9%	9.2%	3.5%	4.7%	6.8%	15.8%
Fusobacteriota	*Fusobacteriaceae*	*Fusobacterium*	36.9%	33.0%	35.0%	36.6%	24.5%	26.7%
Proteobacteria	*Pseudomonadaceae*	*Pseudomonas*	0.0%	0.0%	0.0%	0.0%	0.0%	0.0%
	Succinivibrionaceae	*Anaerobiospirillum*	2.9%	3.5%	6.7%	10.5%	0.4%	0.3%
	Sutterellaceae	*Sutterella*	2.1%	1.3%	2.0%	2.1%	2.7%	2.0%
	Xanthomonadaceae	*Stenotrophomonas*	0.0%	0.0%	0.0%	0.0%	0.0%	0.0%
